# The Hemoglobin-to-Red Cell Distribution Width Ratio to Predict All-Cause Mortality in Patients with Sepsis-Associated Encephalopathy in the MIMIC-IV Database

**DOI:** 10.1155/2022/7141216

**Published:** 2022-12-31

**Authors:** Xiaxuan Huang, Shiqi Yuan, Yitong Ling, Shanyuan Tan, Tao Huang, Hongtao Cheng, Jun Lyu

**Affiliations:** ^1^Department of Neurology, The First Affiliated Hospital of Jinan University, Guangzhou 510630, China; ^2^Department of Clinical Research, The First Affiliated Hospital of Jinan University, Guangzhou 510630, China; ^3^School of Nursing, Jinan University, Guangzhou 510630, China

## Abstract

**Objective:**

The hemoglobin-to-red cell distribution width ratio (HRR) is associated with the prognosis of sepsis-associated encephalopathy (SAE). This study aimed to determine the relationship between HRR and SAE and to clarify the possible mechanism of HRR as a prognostic factor for SAE.

**Methods:**

A multivariate Cox proportional-hazards regression model was used to assess the correlation between HRR and all-cause mortality. Piecewise linear regression and smooth-curve Cox proportional-hazards regression models were used to observe whether there was a nonlinear relationship between HRR and all-cause mortality in SAE.

**Results:**

This study included 8853 patients with SAE. A nonlinear relationship between HRR and SAE was observed through a two-segment regression model. The left inflection point for the HRR threshold was calculated to be 15.54, which was negatively correlated with all-cause mortality (HR = 0.83, 95% CI = 0.76–0.91, p < 0.001). Subgroup analyses revealed significant interactions between white blood cell count, glucose, and patients who received dialysis and HRR. The inverse correlation between HRR and SAE was more pronounced in patients who did not receive vasopressin (HR = 0.91, 95% CI = 0.87–0.96, p < 0.001) than in those who did receive vasopressin (HR = 0.94, 95% CI = 0.88–1.02, p=0.152) and was significantly more pronounced in patients without myocardial infarction (HR = 0.91, 95% CI = 0.88–0.96, p < 0.001) than in those with myocardial infarction (HR = 0.94, 95% CI = 0.87–1.02, p < 0.114).

**Conclusion:**

This large retrospective study found a nonlinear relationship between all-cause mortality and HRR in patients with SAE in intensive care units, with low HRR being inversely associated with increased all-cause mortality in patients with SAE.

## 1. Introduction

Sepsis-associated encephalopathy (SAE), which is a syndrome of diffuse neurological dysfunction, with no associated infectious foci in the central nervous system (CNS). Since host dysregulation does not occur in CNS infections, clear diagnostic criteria are still lacking for clinical practice [1, 2]. SAE occurrence not only affects the autonomic nervous system and neuroendocrine function of patients with general sepsis but also leads to different degrees of cognitive decline, and even accelerates the occurrence and progression of multiple organ failure in sepsis, thus somewhat prolonging the length of stay in intensive care units (ICU). Statistics suggest that the mortality rate caused by SAE in patients with sepsis in ICU has been well over 60% [3]. However, for SAE diagnosis, in addition to the basic screening of the nervous system, EEG, CT, and MRI examinations can only be used to exclude organic CNS lesions. Identifying more risk factors is therefore important for early assessments of survival and prognosis of patients with SAE [4].

Various clinical biomarkers are currently in the diagnosis and prognosis of SAE. Hemoglobin (Hb) is widely applied to the overall anemic condition of patients with sepsis, including nutrition, inflammation, and general condition. There is a very strong correlation between sepsis and encephalopathy caused by shock and prognosis [5]. The red-blood-cell distribution width (RDW) was used as a biochemical index to measure red blood cell (RBC) variation, and increased RDW is not only indicative of malnutrition caused by oversized or undersized RBC, stress response inflammation, dyslipidemia, hypertension, and other abnormal reactions but can also predict early coronary heart disease, cerebral infarction, pulmonary embolism, other cardiovascular and cerebrovascular diseases, and cancer. Previous studies have investigated the effect of RDW on patients with sepsis [6–8]. Hb and RDW are two important clinical parameters. The Hb/RDW ratio (HRR) can especially reflect the early abnormal manifestations of SAE. Meanwhile, no specific SAE biomarker has been found for use in clinical practice [9, 10].

This study therefore utilized data from the Medical Information Mart for Intensive Care-IV (MIMIC-IV) database, which is a generalized and publicly available database that includes baseline data from many patients with critical diseases, to analyze the connection between the HRR and in-hospital deaths due to SAE. The aim was to determine whether HRR can be used as an important specific indicator to distinguish SAE from other inflammatory diseases and to provide new insights for early and effective SAE interventions.

## 2. Materials and Methods

### 2.1. Data Source

This retrospective study was based on the MIMIC-IV database [11, 12]. The MIMIC database is a multiparameter, free-access, open-source, critical care database established by the Massachusetts Institute of Technology. This database is determined to be under the Safe Harbor Provision of the Health Insurance Portability and Accountability Act [13, 14]. It contains detailed clinical information on more than 380,000 patients in ICU during 2008–2019. We completed the collaborating agency training program (certification number: 45351934). The requirement to obtain individual patient consent was waived since the program does not affect clinical care, and all protected health information was anonymized.

### 2.2. Study Population and Data Extraction

In this study, we used structured query language (SQL) via Navicat Premium (version 15.0) to extract meaningful clinical baseline data based on the subject_id of each ICU patient. According to the third internationally recognized definition of sepsis (pyosis-3), sepsis was diagnosed using the latest sepsis 3 criteria, defined as life-threatening infection combined with sequential organ failure assessment (SOFA) score ≥ 2 [15]. SOFA was included according to the abovementioned criteria. Patients were screened according to the following criteria [16]: (1) patients dying or leaving within 24 h since ICU admission; (2) patients with primary brain injury (traumatic brain injury, ischemic stroke, hemorrhagic stroke, epilepsy, or intracranial infection); (3) patients with severe burn and trauma; (4) patients with chronic alcohol or drug abuse; (5) patients with severe liver disease or encephalopathy caused by kidney disease; and (6) patients without a laboratory indicator of HRR.

### 2.3. Sepsis-Associated Encephalopathy

As far as we know, Glasgow Coma Scale (GCS) has been proved to be an important tool to assess the degree of consciousness disturbance in SAE patients. Sepsis patients with GCS ≤ 14 and excluding other unrelated factors can be initially diagnosed as sepsis-related encephalopathy. Therefore, SAE was further defined as sepsis with GCS score ≤14 or delirium diagnosed according to ICD9 code on the first day of admission, and GCS scores assessed before surgery or before sedation and anesthesia will be included as far as possible, and excluded psychiatric disorders, neurological disorders, alcoholism, or trauma from the initial diagnosis of admission [17].

### 2.4. Data Extraction

We used SQL and the PostgreSQL tool (version 15.0) to extract meaningful clinical data. We followed the principle that if a measurement was made multiple times for a patient, we only used the first one. Thus, the first measurement of vital signs and laboratory data within the first 24 hours of ICU admission was included in this study. The initially selected laboratory measurements included ethnicity, age, sex, pregnancy, abrosia, dialysis, heart rate (beats/minute), mean blood pressure (MBP, mmHg), respiratory rate (breaths/minute), temperature (°C), peripheral oxygen saturation (SpO_2_, %), Oxford Acute Severity of Illness Score (OASIS), Simplified Acute Physiology Score II (SAPSII), Logistic Organ Dysfunction System (LODS) score, SOFA score, GCS score, myocardial infarction, congestive heart failure, peripheral vascular disease, chronic pulmonary disease, diabetes, paraplegia, RDW (×10^9^/L), calcium (mg/dL), potassium (mmol/L), Hb (g/L), sodium (mmol/L), glucose (mg/dL), hematocrit (%), platelets (×10^9^/L), the Hb/RDW ratio (HRR) [9, 18, 19], chloride (mmol/L), prothrombin time (PT, seconds), white blood cell (WBC) count (×10^9^/L), anion gap (×10^9^/L), bicarbonate (mmol/L), creatinine (mEq/L), international normalized ratio (INR), norepinephrine, and vasopressin. ICU all-cause mortality referred to in-hospital survival and was used as the outcome of this study.

## 3. Statistical Analysis

All baseline values of continuous variables are expressed as mean ± standard deviation or median values, and the values of categorical variables are expressed as counts and proportions. All continuous variables in this study were compared using t-tests or Wilcoxon rank-sum tests, and the resulting proportions were compared using chi-square or Fisher's exact tests. Differences among HRR tertiles were determined using one-way ANOVA or Kruskal–Wallis H tests. We used a Cox proportional-hazards model to determine the association between HRR and in-hospital mortality in patients with SAE, and developed three different models based on this: an unadjusted model, with no adjustment for covariates; model 1, partially adjusted model (for age and sex only); and model 2, fully adjusted for potential confounders. The results are expressed as hazard ratios (HRs) with 95% confidence intervals (CIs). Results are provided for unadjusted, microadjusted, and fully adjusted analyses.

Furthermore, a generalized additive model was applied to verify the nonlinear relationship between HRR and SAE after it was confirmed using a logarithmic likelihood ratio test (p < 0.05). We used cubic-spline function fitting and the smooth curves of two linear regression models of all-cause mortality in the smooth figure calculation for the HRR threshold. Moreover, a recursive method was used to automatically calculate the inflection points in the fitted graph and infer the likelihood ratio of the optimal model. We analyzed the relationship between HRR and all-cause mortality in patients with SAE between different subgroups to determine whether there were differences in the effect of HRR. The statistical software package R (the R Foundation; https://www.r-project.org) was used to perform data analyses in this study.

## 4. Results

### 4.1. Baseline Results

**Figure 1:**
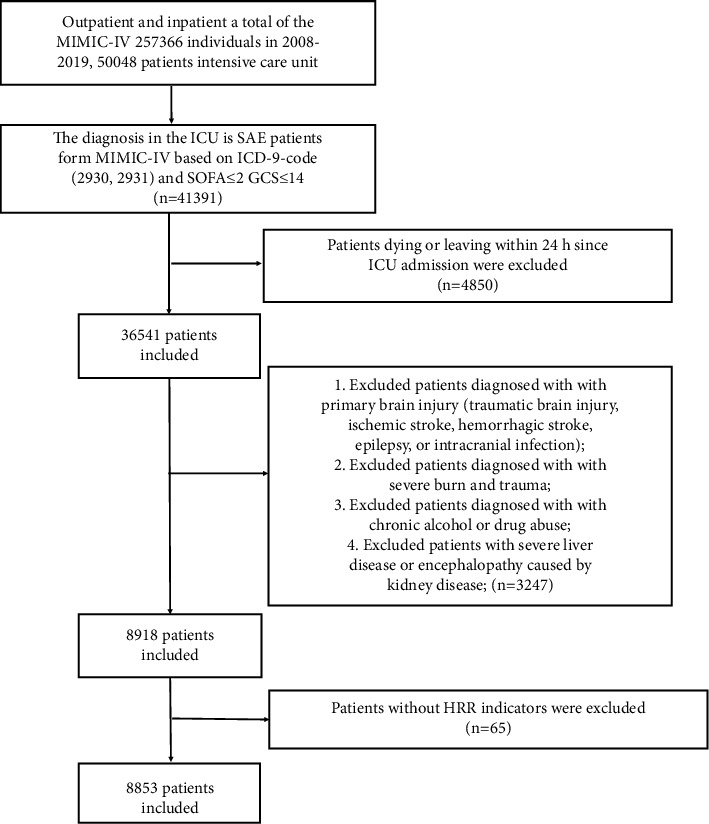
Flowchart of subject screening.

After the above screening criteria, a total of 8853 SAE patients were included in the final cohort, and the inclusion process is shown in Figure 1. In all included population, the median age was 67 years (IQR=55–78 years) and comprised 4007 males (45.30%) and 4845 females (54.70%).. Final outcomes of in-hospital death and survival occurred in 693 (7.80%) and 8159 (92.2%) patients, respectively, with a significant difference (p < 0.001). Quartiles 1 (≥3.98 to <14.00), 2 (≥14.00 to <17.10), and 3 (≥17.10 to ≤28.66) of HRR values comprised 2937, 2967, and 2948 cases, respectively. Differences between groups were assessed using Pearson's chi-square test for categorical variables and t-test for continuous variables. All the variables are listed in Table 1.

### 4.2. HRR and ICU Mortality

We used multivariate Cox proportional-hazards regression models to determine the relationship between HRR and ICU mortality in patients with SAE, with the unadjusted and adjusted models highlighted in Table 2. We observed that low HRR (≥3.98 to <14.00) was significantly associated with an increased all-cause mortality risk in patients with SAE (unadjusted model: HR = 0.87, 95% CI = 0.82–0.92, p < 0.0001; partially adjusted model: HR = 0.85, 95% CI = 0.80–0.90, p < 0.0001; fully adjusted model: HR = 0.86, 95% CI = 0.79–0.93, p=0.0003), while high HRR (≥14.00, <17.10 and ≥17.10, ≤28.66) was not significantly associated with SAE mortality (p > 0.05).

### 4.3. Subgroup Analyses

**Figure 2:**
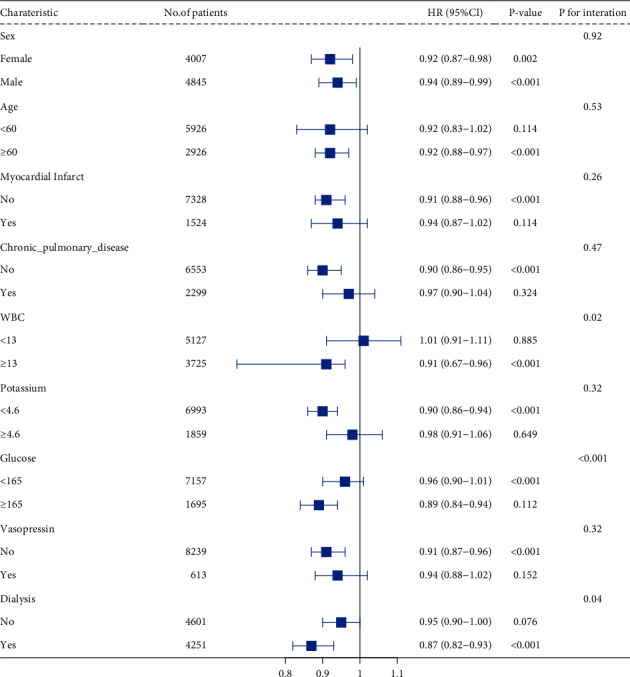
The subgroups of relationship between the HRR and all-cause mortality (horizontal lines indicate the range of the 95% CI and the vertical lines represent the HR).

As shown in Figure 2, we included sex, age, myocardial infarction, chronic lung disease, WBC count, PT, glucose, vasopressin, and dialysis in the subgroup analyses. Except for WBC count, the interaction between glucose and dialysis treatment was significant (interaction p < 0.05), and most of the other formation interactions were not significant (p=0.92–0.78). In the subgroup analyses, age <60 years (HR = 0.92, 95% CI = 0.88–0.97, p < 0.001), WBC ≥ 13 × 10^9^/L (HR = 0.91, 95% CI = 0.67–0.96, p < 0.001), potassium <4.6 mmol/L (HR = 0.90, 95% CI = 0.86–0.94, p < 0.001), glucose ≥165 mg/dL (HR = 0.89, 95% CI = 0.84–0.94, p < 0.001), no myocardial infarction (HR = 0.91, 95% CI = 0.88–0.96, p < 0.0001), and chronic lung disease (HR = 0.90, 95% CI = 0.86–0.95, p < 0.001), as well as these strata of previous dialysis (HR = 0.95, 95% CI = 0.90–1.00, p < 0.001) and vasopressin therapy (HR = 0.91, 95% CI = 0.87–0.96, p < 0.001) had significant interactions. The association between HRR and all-cause mortality in patients with SAE was similar.

### 4.4. Analysis of the Nonlinear Relationship between the HRR and All-Cause Mortality

**Figure 3:**
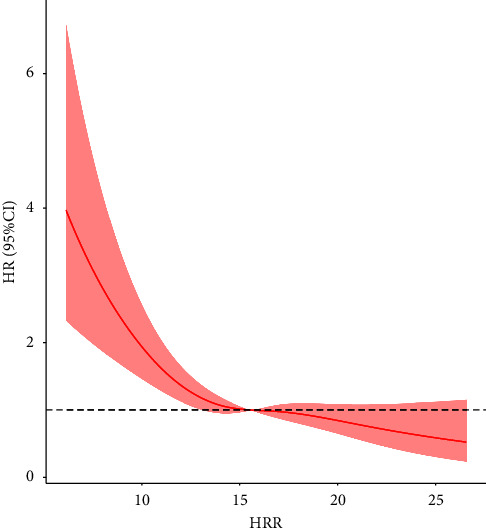
The nonlinear relationship between the HRR and all-cause mortality.

This study found a significant nonlinear relationship between HRR and patients with SAE. We included the following confounding variables that were meaningful to the model: sex, age, sodium, diabetes, calcium, LODS score, SOFA score, GCS score, chloride, myocardial infarction, respiratory rate, temperature, SpO_2_, PT, WBC count, peripheral vascular disease, MBP, paraplegia, norepinephrine, vasopressin, anion gap, bicarbonate, blood urea nitrogen, creatinine, INR, Hb, OASIS, SAPSII, norepinephrine, chronic pulmonary disease, and congestive heart failure.  Figure 3 and Table 3 display the nonlinearity of the relationship between HRR and SAE all-cause mortality. Notably, there was an inverse association between the left inflection-point threshold and all-cause mortality (HR = 0.83, 95% CI 0.76–0.91, p < 0.001), while there was no significant correlation at the right inflection-point threshold (HR = 0.95, 95% CI = 0.91–1.01, p=0.085).

## 5. Discussion

No previous study that we were aware of investigated the connection between HRR and SAE mortality, and this was the first time that a certain relationship was assumed between the ratio of patients with SAE and HRR based on the large MIMIC-IV database. This retrospective study demonstrated a significant nonlinear association between HRR and all-cause mortality in patients with SAE. In addition, even in the presence of certain traditional factors for life-threatening cerebrovascular disease, patients with low HRR were still at a higher risk of death. This study therefore found that lower HRR remained an important predictor of mortality in patients with SAE.

SAE is most common in patients with sepsis in ICU and one of its most serious complications. Its pathogenesis has not yet been fully elucidated, but it may be related to autoimmune injury of the central body inflammatory reaction [20, 21], a blood flow regulating mechanism for the damage caused by cerebral perfusion abnormalities and microvascular dysfunction [22, 23], or a large release of cytokines in sepsis that leads to neurotransmitter imbalance [24]. Polyneuropathy is often more severe in the later stage of SAE development, which not only affects peripheral nerves but also rapidly causes cognitive deficits, personality changes, generalized epilepsy, and other symptoms. Many patients who survive SAE often have a depressive tendency, posttraumatic stress disorder, and other consciousness disorders. The clinical diagnosis of SAE is still performed as an exclusion diagnosis, and laboratory tests have not identified specific SAE biomarkers. It is therefore highly significant to identify specific early warning and diagnostic indicators among the risk factors for SAE in critically ill patients with sepsis for facilitating its early intervention and treatment [25, 26]. HRR has been previously explored as a novel inflammatory marker in many studies on the relationship between HRR and the clinical outcomes of various diseases. There is increasing evidence that HRR is a new prognostic indicator in critically ill patients with malignant diseases. For example, Qin et al. explored the nonlinear relationship between all-cause mortality and HRR value in patients with ischemic stroke [27], and Rahamim et al. found that HRR was an important prognostic tool for predicting mortality in heart failure and the hospitalization of patients with cardiovascular disease [9]. Chi et al. found that low HRR were associated with a dual risk of disease progression or cancer recurrence in patients and used them to predict poor prognosis in patients with cancer [28]. The present single-center, retrospective study confirmed the hypothesis that HRR affects the prognosis of patients with SAE. We found that SAE had an obvious nonlinear relationship with HRR, with a low HRR greatly increasing the risk of a poor prognosis for SAE. A piecewise linear regression model was used in an attempt to calculate the critical inflection point for HRR. It was found that HRR ≤ 15.57 was negatively correlated with all-cause mortality.

The underlying mechanism of HRR predicting the risk of mortality in SAE remains unclear. We attempted to explore the link from a pathophysiological point of view. First, SAE caused by prolonged septic infection often manifests as the nervous system involvement, cerebral hemorrhage, cerebral ischemia, significant hypotension, organ dysfunction, and shock, leading to massive red blood cell loss and failure to timely replantation of bone marrow hematopoiesis, resulting in anemia [29, 30]. Hb somewhat reflects the severity and prognosis of patients with SAE [31]. On the other hand, as another parameter of HRR, RDW is a variation coefficient for measuring the heterogeneity of RBC sizes [32]. Generally, a larger RDW means greater heterogeneity of RBC sizes, or larger differences in shape and size [33, 34]. It can be better explained by the decreased RBC deformation ability under the influence of inflammatory cytokines in patients with sepsis aggravating the abnormal microcirculation in blood perfusion, or the CNS inflammation induced by the oxidative stress response, which is an important indicator of the potential accelerated development of inflammation in SAE [35–37]. The present study was the first to use the HRR as an independent predictive biochemical index, which was found to be significantly more stable than RDW alone, and excluded the influence of traditional factors such as cerebrovascular disease, malignancy, infectious liver disease, severe liver and kidney dysfunction, and trauma. A clear nonlinear relationship was observed between low HRR and poor prognosis in patients with SAE. Previous studies have confirmed the importance of GCS score as a neuroscientific assessment tool to identify patients with SAE at a high risk of short-term mortality early [38, 39]. Therefore, in order to strengthen early interventions for patients with SAE, HRR, and GCS assessments can be used as further prognostic indicators for patients with SAE. We look forward to providing supplementary prognostic information for patients with SAE at earlier stages.

The study had some limitations. First, this large retrospective study had a single-center retrospective design, and the selection of population and covariate factors was somewhat biased, which needs to be addressed by designing prospective multicenter studies in the future. Secondly, the laboratory indicators extracted in this study are relatively few, and the dynamic changes of biochemical indicators are not analyzed in detail. Other undiscovered confounding factors may exist, and it is difficult to further prove the causal association between HRR and SAE all-cause mortality. Therefore, further longitudinal data collection and more advanced statistical methods are needed to conduct causal inference. Thirdly, regarding the cutoff range of HRR values, there has been heterogeneity in previous studies on different critical diseases, and there is no evident standard cutoff value for this ratio in clinical practice. Finally, long-term follow-up events were not analyzed, instead the focus being on in-hospital all-cause mortality among patients with SAE. We look forward to further research with more rigor and depth, especially to address these issues through studies with a multicenter, follow-up, and prospective design.

## 6. Conclusion

In summary, this large retrospective study has identified a nonlinear association between all-cause mortality and HRR in patients with SAE, with a low HRR being negatively associated with increased ICU mortality in patients with SAE.

## Figures and Tables

**Table 1:** Baseline characteristics.

	Overall	Q1 (3.98–14.00)	Q2 (14.00–17.10)	Q3 (17.10–28.66)	*P* value
N	8852	2937	2967	2948	
*Characteristic ethnicity (%)*					
Other	2776 (31.4%)	868 (29.6%)	936 (31.5%)	972 (33.0%)	0.018
White	6076 (68.6%)	2069 (70.4%)	2031 (68.5%)	1976 (67.0%)	
Age (year)	67.00 (55.00, 78.00)	71.00 (60.00, 81.00)	68.00 (57.00, 79.00)	61.00 (46.00, 73.00)	<0.001
*Sex, n (%)*					
Female	4007 (45.3%)	1691 (57.6%)	1400 (47.2%)	916 (31.1%)	<0.001
Male	4845 (54.7%)	1246 (42.4%)	1567 (52.8%)	2032 (68.9%)	
*Pregnant (%)*					
No	8006 (90.4%)	2603 (88.6%)	2691 (90.7%)	2712 (92.0%)	<0.001
Yes	846 (9.6%)	334 (11.4%)	276 (9.3%)	236 (8.0%)	
*Abrosia (%)*					
No	20 (0.2%)	7 (0.2%)	8 (0.3%)	5 (0.2%)	0.71
Yes	8832 (99.8%)	2930 (99.8%)	2959 (99.7%)	2943 (99.8%)	
*Dialysis (%)*					
No	4601 (52.0%)	1544 (52.6%)	1563 (52.7%)	1494 (50.7%)	0.224
Yes	4251 (48.0%)	1393 (47.4%)	1404 (47.3%)	1454 (49.3%)	
*Ventilation (%)*					
No	928 (10.5%)	259 (8.8%)	229 (7.7%)	440 (14.9%)	<0.001
Yes	7924 (89.5%)	2678 (91.2%)	2738 (92.3%)	2508 (85.1%)	
*Vital signs*					
Heart rate (beats/minute)	86.00 (76.50, 97.50)	86.50 (77.00, 98.50)	85.50 (76.50, 96.50)	85.00 (75.50, 97.00)	<0.001
MBP (mmHg)	79.00 (72.50, 86.00)	77.00 (70.50, 85.00)	78.50 (73.00, 85.50)	80.50 (74.00, 88.50)	<0.001
Respiratory rate (breath/minute)	19.50 (17.00, 22.25)	20.00 (17.50, 23.00)	19.50 (17.00, 22.00)	19.50 (17.00, 22.00)	<0.001
Temperature (°C)	36.84 (0.62)	36.81 (0.63)	36.82 (0.57)	36.90 (0.64)	<0.001
SpO_2_ (%)	96.50 (95.00, 97.50)	96.00 (95.00, 97.50)	96.50 (95.00, 97.50)	96.50 (95.00, 97.50)	0.194
*Scoring systems*					
OASIS	34.00 (28.00, 40.00)	35.00 (29.00, 41.00)	34.00 (28.00, 40.00)	32.00 (26.00, 38.00)	<0.001
SAPSII	35.00 (27.00, 44.00)	37.00 (30.00, 46.00)	35.00 (28.00, 44.00)	31.00 (24.00, 41.00)	<0.001
LODS	5.00 (4.00, 8.00)	6.00 (4.00, 9.00)	5.00 (4.00, 8.00)	5.00 (3.00, 7.00)	<0.001
SOFA	5.00 (3.00, 7.00)	5.00 (3.00, 8.00)	5.00 (3.00, 7.00)	4.00 (3.00, 7.00)	<0.001
GCS	13.00 (10.00, 14.00)	13.00 (9.00, 14.00)	13.00 (10.00, 14.00)	13.00 (9.00, 14.00)	0.677
*Comorbidities, n (%)*					
* Myocardial infarction*					
No	7328 (82.8%)	2388 (81.3%)	2467 (83.1%)	2473 (83.9%)	0.026
Yes	1524 (17.2%)	549 (18.7%)	500 (16.9%)	475 (16.1%)	
* Congestive heart failure*					
No	6609 (74.7%)	1957 (66.6%)	2214 (74.6%)	2438 (82.7%)	<0.001
Yes	2243 (25.3%)	980 (33.4%)	753 (25.4%)	510 (17.3%)	
* Peripheral vascular disease*					
No	7895 (89.2%)	2543 (86.6%)	2635 (88.8%)	2717 (92.2%)	<0.001
Yes	957 (10.8%)	394 (13.4%)	332 (11.2%)	231 (7.8%)	
* Chronic pulmonary disease*					
No	6553 (74.0%)	1993 (67.9%)	2196 (74.0%)	2364 (80.2%)	<0.001
Yes	2299 (26.0%)	944 (32.1%)	771 (26.0%)	584 (19.8%)	
* Diabetes*					
No	8477 (95.8%)	2779 (94.6%)	2828 (95.3%)	2870 (97.4%)	<0.001
Yes	375 (4.2%)	158 (5.4%)	139 (4.7%)	78 (2.6%)	
* Paraplegia*					
No	8652 (97.7%)	2859 (97.3%)	2913 (98.2%)	2880 (97.7%)	0.095
Yes	200 (2.3%)	78 (2.7%)	54 (1.8%)	68 (2.3%)	
*Laboratory parameters*					
Calcium (mg/dL)	8.25 (7.80, 8.70)	8.10 (7.70, 8.50)	8.20 (7.80, 8.60)	8.45 (8.00, 8.90)	<0.001
Potassium (mmol/L)	4.15 (3.90, 4.50)	4.20 (3.90, 4.55)	4.20 (3.90, 4.50)	4.15 (3.85, 4.45)	0.002
Sodium (mmol/L)	138.50 (136.50, 141.00)	139.00 (136.00, 141.00)	138.50 (136.50, 140.50)	139.00 (137.00, 141.00)	0.138
Glucose (mg/dL)	127.00 (108.50, 153.00)	126.50 (107.00, 152.50)	127.00 (109.00, 152.50)	128.00 (110.00, 154.62)	0.004
Hematocrit (%)	32.60 (28.85, 36.71)	27.85 (25.90, 30.10)	32.20 (30.15, 34.45)	38.05 (35.40, 40.95)	<0.001
PLT (10^9^/L)	193.00 (145.50, 255.50)	192.00 (136.50, 281.50)	182.00 (139.00, 241.50)	202.50 (161.00, 253.00)	<0.001
Hemoglobin (g/L)	21.60 (19.10, 24.50)	18.20 (16.80, 19.50)	21.50 (20.30, 22.80)	25.60 (24.00, 27.50)	<0.001
RDW (10^9^/L)	13.90 (13.10, 15.00)	15.30 (14.20, 16.70)	13.80 (13.20, 14.50)	13.20 (12.70, 13.72)	<0.001
Chloride (mmol/L)	105.50 (102.00, 108.50)	105.50 (101.50, 109.00)	106.00 (102.50, 108.50)	105.00 (102.00, 107.00)	<0.001
PT (seconds)	13.85 (12.55, 15.50)	14.60 (13.15, 16.65)	13.90 (12.70, 15.38)	13.10 (12.00, 14.45)	<0.001
WBC (10^9^/L)	12.00 (9.10, 15.60)	11.70 (8.70, 15.40)	12.05 (9.20, 15.62)	12.25 (9.40, 15.70)	<0.001
Anion gap (mmol/L)	13.00 (11.50, 15.50)	13.00 (11.00, 15.00)	13.00 (11.00, 15.00)	14.00 (12.00, 16.00)	<0.001
Bicarbonate (mmol/L)	23.67 (4.32)	23.69 (4.79)	23.72 (4.11)	23.61 (4.02)	0.584
Bun (mg/dL)	16.50 (12.50, 24.00)	19.00 (13.00, 29.50)	16.50 (12.50, 22.50)	15.50 (12.00, 21.00)	<0.001
Creatinine (mEq/L)	0.90 (0.70, 1.15)	0.90 (0.70, 1.20)	0.85 (0.70, 1.10)	0.90 (0.75, 1.10)	<0.001
INR	1.25 (1.10, 1.40)	1.30 (1.20, 1.50)	1.25 (1.15, 1.40)	1.20 (1.10, 1.30)	<0.001
*Treatment, n (%)*					
* Norepinephrine*					
No	6926 (78.2%)	2094 (71.3%)	2360 (79.5%)	2472 (83.9%)	<0.001
Yes	1926 (21.8%)	843 (28.7%)	607 (20.5%)	476 (16.1%)	
* Vasopressin*					
No	8239 (93.1%)	2663 (90.7%)	2770 (93.4%)	2806 (95.2%)	<0.001
Yes	613 (6.9%)	274 (9.3%)	197 (6.6%)	142 (4.8%)	
ICU death, *n* (%)	0.08 (0.27)	0.11 (0.31)	0.07 (0.25)	0.06 (0.24)	<0.001
No	8159 (92.2%)	2618 (89.1%)	2766 (93.2%)	2775 (94.1)	<0.001
Yes	693 (7.8%)	319 (10.9%)	201 (6.8%)	201 (5.9%)	

**Table 2:** Factors independently correlated to all-cause mortality in HRR by univariate analysis.

HRR (quartile)	*Nonadjusted*		*Adjusted model I*		*Adjusted model II*	
	HR (95% CIs)	*P* value	HR (95% CIs)	*P* value	HR (95% CIs)	*P* value
≥3.98, <14.00	0.87 (0.82–0.92)	<0.0001	0.85 (0.80–0.90)	<0.0001	0.86 (0.79–0.93)	0.0003
≥14.00, <17.10	1.01 (0.86–1.17)	0.9202	1.08 (0.92–1.26)	0.3604	1.05 (0.87–1.27)	0.5869
≥17.10, ≤28.66	0.96 (0.89–1.03)	0.2725	1.02 (0.94–1.10)	0.6063	0.96 (0.84–1.08)	0.4656

(nonadjusted model; adjusted model I: adjusted for sex and age; adjusted model II: sex, age, sodium, diabetes, calcium, LODS score, SOFA score, GCS score, chloride, myocardial infarction, respiratory rate, temperature, SpO_2_, PT, WBC count, peripheral vascular disease, MBP, paraplegia, norepinephrine, vasopressin, anion gap, bicarbonate, blood urea nitrogen, creatinine, INR, Hb, OASIS, SAPSII, norepinephrine, chronic pulmonary disease, and congestive heart failure).

**Table 3:** Analysis of nonlinear relationship between the HRR and all-cause mortality.

Inflection point of HRR	HR (95% CI)	*P* value
<15.57	0.83 (0.76–0.91)	<0.0001
≥15.57	0.95 (0.91–1.01)	0.085

## Data Availability

Publicly available datasets are analyzed in this study. The data are available on the MIMIC-IV website at https://mimic-iv.mit.edu/.
